# An Observation Study of Caries Experience and Potential Risk Assessments among Disabled Individuals Living in an Institutional Rehabilitation Centre

**DOI:** 10.3390/life14050605

**Published:** 2024-05-09

**Authors:** Abdullah Ali H. Alzahrani, Nagesh Bhat

**Affiliations:** 1 Dental Health Department, Faculty of Applied Medical Sciences, Al-Baha University, Al-Baha 65731, Saudi Arabia; 2 Department of Preventive Dental Sciences, School of Dentistry, Al-Baha University, Al-Baha 65731, Saudi Arabia; n.bhat@bu.edu.sa.com

**Keywords:** disabled adults, oral health, special care dentistry, dental public health, cariogram

## Abstract

The aim of this study was to conduct salivary, microbiological, and caries risk assessments in relation to caries experience among individuals with intellectual disability in an institutional center in the Al-Baha region, Saudi Arabia. A cross-sectional study was conducted among 89 patients residing in special care homes in the Al-Baha region, Saudi Arabia, from October 2023 to February 2024. The demographic details of all participants were recorded. Clinical oral examinations were performed for the decayed, missing, or filled teeth (DMFT) and plaque indices. Salivary and microbiological assessments were also carried out. The mean age of the study sample was 30.11 ± 4.39 years, and the mean duration of years spent residing in the facility was 26.49 ± 4.66. There was no significant difference observed across plaque scores, *S. mutans* colony count, salivary rate, pH, DFMT, and caries experience when they were compared across the levels of severity of intellectual disability. Statistically significant differences were observed across diet score, circumstance score, and chances to avoid caries and were found to be correlated with the severity of intellectual disability (*p* = 0.001, *p* = 0.001, and *p* = 0.002), respectively. The cariogram revealed that participants in this study had poor oral health status, with participants with severe intellectual disability having higher diet scores, frequency scores, and susceptibility scores; hence lesser chances to avoid dental caries. Regular dental check-ups, including cleanings and other treatments if necessary, seem to be fundamental to prevent dental issues and maintain healthy teeth and gums for this group of people. Developing interventions that focus on improving oral health status among intellectually disabled individuals may be recommended to ensure the optimum level of support and reduce the burden of dental decay among those individuals.

## 1. Introduction

In both legal and clinical contexts, disability refers to a degree of functional impairment that is significant enough to impede major life activities. A physical disadvantage impacts a person’s ability to carry out daily tasks. Intellectual disorders must result in either distress or functional impairment as part of their definition; yet, not all mental disorders lead to disability, and not all disabilities are caused by psychiatric conditions [1].

Intelligence is a broad and complex construct encompassing various cognitive abilities such as reasoning, planning, problem-solving, abstract thinking, comprehension of complex ideas, efficient learning, and learning from experience. It is a multidimensional trait that involves both innate potential and environmental influences. Intellectual disability, or cognitive impairment, is characterized by significant limitations in intellectual functioning and adaptive behavior. 

In the past, intelligence has been defined by standardized measures of intelligence, with an intelligence quotient (IQ) score of less than 70 indicating significant cognitive deficits [2]. However, this definition has been criticized for being too narrow and failing to capture the full range of abilities and limitations of individuals with intellectual disabilities. In addition to cognitive deficits, intellectual disability is also characterized by significant impairments in adaptive skills such as communication, self-care, social interaction, and independent living. These limitations can significantly affect an individual’s ability to function independently, meaning that they may require ongoing support and assistance from caregivers and professionals. Overall, understanding the complex nature of intelligence and intellectual disability is essential for improving the quality of life and promoting the inclusion of individuals with cognitive impairments in society [3]. 

Individuals of all ages and social classes are affected, but adults with disabilities tend to have poorer health and receive less care than the general population [4,5]. Oral health determines the overall health of the body [6]. However, individuals with disabilities may face a range of challenges when it comes to maintaining optimal oral health. Various conditions, such as intellectual disability, developmental or physical disability, cerebral palsy, craniofacial anomalies, and epilepsy, can negatively impact oral health. 

Oral health issues are more prevalent in people with disabilities, and a wide range of factors can contribute to this, including their impairment, underlying medical conditions, social circumstances, medications, lack of access to oral health care, or even a lack of awareness from parents or caregivers [7,8]. It is, therefore, crucial to be aware of these factors and take steps to ensure that individuals with disabilities receive the necessary oral health care and support to maintain good oral health and overall well-being [9].

Dental caries continues to be a significant problem for both children and adults and a dental public health concern worldwide [10]. Research indicates that the rate of dental caries in Saudi Arabia is relatively high, particularly among adolescents. A systematic review found that 80% of Saudi children suffer from dental caries in their primary teeth, while 70% have it in their permanent teeth. The study also found that the respective mean decayed, missing, or filled teeth (DMFT) index scores are 5.0 ± 3.5 [11,12]. Dental caries has been reported in 99% of a sample of 255 Saudi adults with mean DMFT index score 9.1 ± 5.6 [13]. Furthermore, dental caries has been shown to be highly prevalent among Saudi children with and without intellectual disabilities 77% and 86%, respectively. It was concluded that not only did Saudi children with intellectual disability have significantly higher dental discomfort scores than non-disabled children; but also their decayed teeth scores were higher [14]. All those studies may significantly highlight the high prevalence of caries among different groups in the Saudi population. 

Accurate caries risk assessment is essential to identify individuals at greater risk of developing caries in the future. This process aids in assessing the probability of new caries lesions that may occur in the future [15]. Dental caries is a disease caused by bacteria, plaque on teeth, eating habits, and more [16,17]. Preventive measures vary based on individual factors. It is necessary to achieve a balance between pathological and preventive factors, which can lead to tooth decay and loss when pathological factors prevail [18,19]. Henceforth, managing dental caries can be complex, especially for patients with multiple pathologic caries risk factors. This is particularly true for patients with intellectual disability.

It has been observed that the occurrence of tooth decay is not necessarily more significant among individuals with disabilities than it is among the general population. However, numerous studies have found that people with disabilities tend to have more untreated cavities, indicating a higher need for dental treatment and lower levels of oral hygiene and periodontal health than in those without disabilities [20,21,22]. 

Despite significant advancements in dental technology and treatment quality over the past decade, there remain substantial and unjust disparities in the quantity and quality of dental care provided to individuals with disabilities. This study aimed to explore salivary, microbiological, and caries risk assessments in relation to caries experience among individuals with intellectual disability in the institutional center in the Al-Baha region, Saudi Arabia.

## 2. Materials and Methods

### 2.1. Participants and Settings

A cross-sectional observational study was conducted among patients who resided in the special care home in Al Baha state, Saudi Arabia, from October 2023 to February 2024. 

The level of intellectual disability diagnosis was obtained from the medical records of individuals receiving special home care. The diagnosis was categorized as mild, moderate, or severe according to the International Classification of Diseases-10. Each individual with a disability underwent examination and assessment by a designated and qualified hospital, and the results were recorded in their medical records. 

A comprehensive dental assessment was conducted using a specialized form that included a structured interview covering related comorbidities, diet frequency, and fluoride content in toothpaste. To determine the patient’s caries risk profile, the cariogram model was employed, taking into account caries experience, related diseases, diet content, diet frequency, plaque amount, mutans streptococci, fluoride program, saliva secretion rate, saliva buffering capacity, and clinical judgment [23]. 

The patient’s risk was categorized as “shallow risk” (81–100% chance to avoid caries), “low risk” (61–80% chance to avoid caries), “moderate risk” (41–60% chance to avoid caries), “high risk” (21–40% chance to avoid caries), or “very high risk” (0–20% chance to avoid caries). Moreover, Enhancing the Quality and Transparency Of Health Research (EQUATOR) guidelines were applied in the present study. 

### 2.2. Inclusion and Exlusion Criteria 

**Figure 1:**
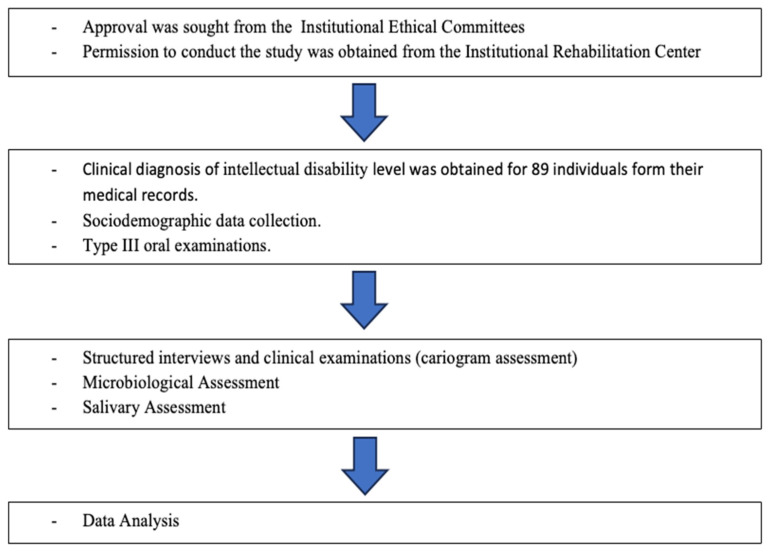
The Study’s Methodology Flowchart.

Individuals of both genders aged 18 years and above who were seeking treatment for intellectual disability were included. Those who refused to participate or sign the consent form were excluded from the study. Figure 1 describes the methodology flowchart of this study.

### 2.3. Sample Size Calculation 

Sample size was calculated based on the pilot study. The prevalence of periodontal disease among adults was found to be 94% using the formula four p × q/d^2^. The final estimated sample size was 87 individuals, 80% power, and a 5% precision level. 

### 2.4. Dental Examination

Before the study began, two examiners were trained on data collection methods. The inter-examiner variability was assessed, resulting in a weighted kappa statistic of 0.91. With sufficient natural lighting, a clinical oral examination of Type III was conducted using a mouth mirror and community periodontal index probe. The clinical examination evaluated plaque and DMFT scores was conducted using the World Health Organization guidelines [24].

### 2.5. Microbial Assessment and S. mutans

Microbiological assessment was performed, including an *S. mutans* colony count. Supragingival plaque was collected using a sterile swab and placed in a tube of phosphate-buffered saline, then taken to the lab. The plaque was spread on mitis salivarius bacitracin agar and incubated at (95% N2 and 5% CO2) at 37 °C for 48 h. Colonies with *S. mutans* characteristics were counted and identified based on opaque, firm, and easily displaced colonies surrounded by a white halo with a droplet of polysaccharide on top.

### 2.6. Salivary and pH Sampling

Salivary and pH assessments were performed, including salivary flow rate and saliva buffer capacity. Plaque from the interproximal sites of the molar area was collected using the plaque collector of the plaque pH indicator kit. To reduce the risk of contamination with saliva, gentle air-drying was performed before the plaque sample was collected. The plaque collector, with the attached plaque, was dipped in plaque pH indicator solution for 1 s and left to ferment for 5 min. After 5 min, the pH was measured by checking the color and comparing it with the chart on the dispensing dish supplied with the plaque pH indicator kit.

### 2.7. Statistical Analysis 

Statistical analysis was conducted using the Statistical Package for the Social Sciences (SPSS) software version 20.0 (IBM, Armonk, NY, USA), and two-tailed *p* < 0.05 was considered to indicate statistical significance. Frequency distribution was used for descriptive analysis. A chi-square test was used to measure categorical variables. One-way ANOVA was used for intergroup comparison, followed by a post hoc Bonferroni correction test.

## 3. Results

### 3.1. Demographic Characteristics

The study sample comprised 89 adults, all of whom had intellectual disability. The mean age of the sample was 30.11 ± 4.39 years, and the mean duration of years residing in the facility was 26.49 ± 4.66. The majority of the study participants (58.4%) were female. 

There was a difference in the distribution of participants according to the type of intellectual disability, where 43.8% were classed as mild, 29.2% were classed as moderate, and 27% were classed as having severe intellectual disability. Physical disability was seen to be an additional disability. Approximately 44.9% of the participants had malocclusion, and all of them were on medication. Table 1 describes participants’ demographic characteristics.

### 3.2. Diet, Salivary and Bacterial Parameters in relation to Severity of Intellectual Disability

The results of this study showed that the means of the plaque score, *S. mutans* colony count (CFU/10^3^), salivary rate, pH score, and buffer capacity for the participants were (1.92 ± 0.41), (2785.26 ± 414.97), (1.99 ± 1.12), (7.49 ± 1.02), (8.70 ± 3.80), respectively. There was no significant difference across these parameters when they were compared across the levels of severity of intellectual disability. Table 2 illustrates the comparison of diet, salivary and bacterial parameters according to severity of the participants’ intellectual disability.

### 3.3. Cariogram and Severity of Intellectual Disability

Caries risk assessment was conducted using a cariogram model [23]. The mean diet score of participants was 10.62 ± 3.06. The difference was statistically significant (*p* = 0.001), with a higher rate being reported for participants with severe intellectual disability and the lowest rate reported for participants with mild intellectual disability. The means of bacterial count and susceptibility scores of participants were (15.92 ± 3.42) and (31.64 ± 7.05), respectively; indicating comparable values across various severity categories of intellectual disability. 

The participants’ circumstance score was (4.00 ± 1.62). The circumstance score for having caries for participants in the mild category was significantly lower than the circumstance score for participants in the severe category (*p* = 0.001). Moreover, participants’ chances of avoiding caries were 37.87 ± 8.63, indicating a significant difference among the three categories (*p* = 0.002). The chances of avoiding caries for patients with mild intellectual disability were significantly higher than for participants in the severe categories. Table 3 shows a comparison of cariograms according to the severity of the participants’ intellectual disability. 

### 3.4. Risk Category and Severity of Intellectual Disability

Among 89 individuals who participated in this study, only two participants belonged to the very-low-risk category. On comparison, a significant observation was made regarding the higher number of participants in the low-risk category, as they were found to have mild intellectual disability (*p* = 0.021). Table 4 describes distribution of participants according to risk category and severity of intellectual disability. 

The mean scores of caries experience and DMFT of participants were (1.95 ± 0.32) and (10.97 ± 4.59), respectively. No significant differences were observed for both caries experience and DMFT scores across various severity categories of intellectual disability. Table 5 shows a comparison of participants DMFT and caries experience according to risk category.

## 4. Discussion

Managing dental caries effectively involves assessing the individual’s risk of caries and creating a customized treatment plan based on the data gathered during the risk assessment. It is important to regularly evaluate the risk of future dental caries to monitor any changes in oral health over time. Health professionals must provide equal-quality services to people with disabilities based on free and informed consent, and accessibility must be ensured [25,26,27]. This study aimed to explore salivary, microbiological, and caries risk assessments in relation to caries experience among individuals with intellectual disability in the institutional center in the Al-Baha region, Saudi Arabia.

In the present study, the DMFT score was 10.97 ± 4.59, indicating no significant difference across various categories of intellectual disability. This was in accordance with the findings from Germany, where the mean DMFT of the study participants ranged from 9.5 to 10.9 [28]. However, other researchers found that dental examinations of 158 patients recorded the lower DMFT score as 4.90 ± 4.63 and found no statistical significance (*p* = 0.142) in the mean DMFT score among the various types of disability [29]. It could be highlighted that the variation in the DMFT scores across different populations may be attributed to the different age groups, socioeconomic statuses, and methodologies used for assessing the DMFT index among those populations. 

For the study participants, the mean plaque score was 1.92 ± 0.41, with no statistical difference. This is in agreement with other research that reported the mean plaque scores among intellectually disabled people was not statistically significant (1.16 ± 0.58) [29]. On the other hand, this study revealed that the mean *S. mutans* colony count (CFU/10^3^) among intellectually disabled individuals was 2785.50 ± 414.97. Likewise, Katge F. et al. (2015) reported that the mean number of *S. mutans* colony-forming units found was 2.961 × 104 among disabled people [30]. It could be underlined that for the predictive threshold of salivary mutans streptococci, no absolute values for high or low values have been established. These findings highlight that in patients with intellectual disabilities, the amount of bacteria in the dental biofilm seems higher. 

The findings of this study showed that the means salivary rate and buffer capacity were 1.99 ± 1.12 and 8.70 ± 3.80; indicating no significant difference across these parameters when they were compared across severity of intellectual disability. Similarly, other research had reported no significant difference between salivary rate flow and buffer capacity in relation to the severity of intellectual disability [31,32]. Moreover, the mean pH score in the present study was 7.49 ± 1.02. Other research in the dental literature recorded a mean pH score that ranged from 6 to more than 6.2 [30,31]. Studies suggest that people prone to dental caries have higher acid production and lower salivary buffering capacity. This can lead to impaired neutralization of plaque acids and reduced remineralization of early enamel lesions, which increases the risk of caries development. On the other hand, a high salivary buffering capacity is associated with lower caries levels [33]. The mouth contains a balance between the number of free bacteria in saliva and those attached to the teeth or oral epithelial cells. A low rate of salivary flow increases the risk of developing caries. Factors that can cause reduced secretion of saliva include medication, pathological changes in the salivary glands, and age. It is considered a potential risk factor when the unstimulated salivary flow rate is lower than 0.30 mL/min, and the stimulated salivary flow rate is lower than 0.7 mL/min [33,34].

Caries risk assessment was conducted using a cariogram model. The diet score was statistically significant for the study’s participants (10.62 ± 3.06, *p* = 0.001), with a higher rate being reported for patients with severe intellectual disability and the lowest rate reported for participants with mild intellectual disability. Likewise, other research found that the highest diet scores were found amongst the severely intellectually disabled people, but the difference was not statistically significant [31]. This might be attributed to the settings where the study was conducted, as it involved intellectually disabled individuals at ab institutionalized rehabilitation center; thus, they may be following “standardized” diets that have been served at the center. Furthermore, the bacterial count and susceptibility mean scores of participants in the current study were 15.92 ± 3.42 and 31.64 ± 7.05, respectively, indicating no significant association across various severity categories of intellectual disability. In contrast, another study reported a significant relationship between bacterial count and susceptibility, with a higher rate being reported for the group with severe intellectual disability group and a lower rate reported among the group with mild intellectual disability (*p* < 0.001 and *p* = 0.02), respectively [31,35]. This might be attributed to the differences in the settings where the studies were conducted and the age groups of the participants included in the studies. 

The circumstance score for having caries for the participants in the mild intellectual disability category was significantly lower than the circumstance score for patients in the severe intellectual disability category (4.00 ± 1.62, *p* = 0.001). Moreover, the chances of avoiding caries for the mild intellectual disability group in the present study was significantly higher than that for the participants in the severe categories (37.87 ± 8.63, *p* = 0.002). This is consistent with other research that found a statistically significant association between chances of avoiding caries and the severity of intellectual disability (*p* = 0.009) [31]. 

Individuals with disabilities often experience severe psychological, physical, and intellectual challenges, which can negatively impact their oral health. Inadequate dental care or poor dental public health measures can exacerbate these issues [36,37,38,39]. Due to anatomical malformations of the orofacial cavity and uncooperative behavior, disabled people often require the assistance of parents or caretakers to maintain good oral hygiene. Poor oral hygiene and inadequate tooth brushing are the most significant risk factors for dental caries and periodontal disease among disabled individuals [40,41,42]. The occurrence of these patterns has been associated with several aspects. The first is the key role of caregivers in providing oral health support and the significance of effective educational interventions for caregivers [21]. The second relates to challenges in interactions between dentists, caregivers, and disabled individuals. The last factor is caregivers’ lack of awareness regarding people’s dental treatment needs [43]. Nevertheless, interestingly, tooth brushing training with an augmented reality device and a smart toothbrush has shown to be more useful and effective than visual training materials among intellectual disabled individuals in Korea [44]. 

This unique population with developmental disabilities and differing healthcare needs often faces difficulties in maintaining their oral health. Even for typical individuals, daily oral care can be challenging for several reasons, such as a lack of knowledge of proper oral hygiene practices or difficulty in following an oral healthcare routine. Therefore, providing disabled individuals and their parents or caregivers with the necessary guidance and training might be an essential aspect to ensure optimal oral health. Additionally, regular dental check-ups, including cleanings and other treatments if necessary, seem to be fundamental to prevent dental issues and maintain healthy teeth and gums for this group of people. Developing interventions that focus on improving oral health status among intellectually disabled individuals may be advocated to ensure the optimum level of support and reduce the burden of dental decay among those disabled individuals.

Like any other research, this study had some limitations. First, the study only looked at individuals with intellectual disabilities in one care setting, thus the findings might not apply to other groups or places, which may limit the generalizability of the study findings. Second, there was potential inhibition of the ability to assess causative associations between the variables of the study due to the study’s cross-sectional design. However, the focus of the study was on providing insight into salivary, microbiological, and caries risk assessments in relation to caries experience among individuals with intellectual disability in the institutional center in the Al-Baha region, Saudi Arabia, rather than on measuring causal relationships. Finally, longitudinal studies with intervention and longer follow-up are needed to gauge the services rendered to this intellectually disabled population.

## 5. Conclusions

Participants of this study had poor oral health status, with individuals with severe intellectual disability having higher diet scores, frequency scores, and susceptibility scores, and hence lesser chances to avoid dental caries as assessed by a cariogram. There were no significant differences across plaque scores; *S. mutans* colony count, salivary rate, pH, DFMT, and caries experience when they were compared in terms of the severity of intellectual disability. However, statistically significant differences were seen across diet score, circumstance score, and chances of avoiding caries in association with the severity of intellectual disability (*p* = 0.001, *p* = 0.001, and *p* = 0.002).

## Figures and Tables

**Table 1:** Demographic characteristics of the study participants.

Variable		N (%)
Age Group	18–25 years	1 (1.1)
	26–30 years	58 (65.2)
	31–35 years	21 (23.6)
	36 years and above	9 (10.1)
Gender	Male	37 (41.6)
	Female	52 (58.4)
Level of Intellectual Disability	Mild	39 (43.8)
	Moderate	26 (29.2)
	Severe	24 (27)
Type of Additional Disability	None	45 (50.6)
	Hearing	1 (1.1)
	Physical	39 (43.8)
	Visual	4 (4.5)
Medication	Yes	89 (100)
	No	0 (0)
Comorbidity	Yes	59 (66.3)
	No	30 (33.7)

**Table 2:** Comparison of diet, salivary and bacterial parameters according to severity of the participants’ intellectual disability.

Variable		n	Mean ± Std. Deviation (SD)	95% Confidence Interval for Mean		Minimum	Maximum	*p* Value
				Lower Bound	Upper Bound			
Plaque scores	Mild	39	1.84 ± 0.36	1.72	1.96	1.42	2.88	0.245
	Moderate	26	2.00 ± 0.46	1.81	2.18	1.41	3.19	
	Severe	24	1.98 ± 0.42	1.80	2.15	1.48	2.85	
	Total	89	1.92 ± 0.41	1.84	2.01	1.41	3.19	
*S. mutans* Colony Count (CFU/10^3^)	Mild	39	2856.36 ± 430.73	2716.73	2995.99	1836.00	3630.00	0.308
	Moderate	26	2696.85 ± 406.43	2532.69	2861.00	1696.00	3240.00	
	Severe	24	2765.50 ± 393.66	2599.27	2931.73	1768.00	3600.00	
	Total	89	2785.26 ± 414.97	2697.84	2872.67	1696.00	3630.00	
Salivary Rate (mL/min)	Mild	39	1.80 ± 0.88	1.52	2.09	0.60	4.80	0.115
	Moderate	26	1.91 ± 1.12	1.46	2.36	0.89	5.20	
	Severe	24	2.39 ± 1.39	1.80	2.98	1.00	5.60	
	Total	89	1.99 ± 1.12	1.76	2.23	0.60	5.60	
pH	Mild	39	7.46 ± 0.91	7.16	7.75	5.60	10.20	0.090
	Moderate	26	7.22 ± 1.07	6.79	7.65	5.89	9.70	
	Severe	24	7.85 ± 1.07	7.40	8.30	5.90	10.30	
	Total	89	7.49 ± 1.02	7.28	7.71	5.60	10.30	
Buffer Capacity (mol/L)	Mild	39	9.41 ± 3.88	8.15	10.67	5.00	18.00	0.163
	Moderate	26	7.58 ± 3.19	6.29	8.87	2.00	18.00	
	Severe	24	8.75 ± 4.13	7.01	10.49	5.00	21.00	
	Total	89	8.70 ± 3.80	7.90	9.50	2.00	21.00	

One-way ANOVA. Level of significance set at *p* < 0.05, * *p* = statistically significant.

**Table 3:** Comparison of cariogram according to severity of the participants’ intellectual disability.

Variable		n	Mean ± Std. Deviation (SD)	95% Confidence Interval for Mean		Minimum	Maximum	*p* Value
				Lower Bound	Upper Bound			
Diet Score	Mild	39	9.44 ± 2.67	8.57	10.30	5.00	16.00	0.001 * Mild < Severe
	Moderate	26	10.00 ± 2.81	8.86	11.14	5.00	15.00	
	Severe	24	13.21 ± 2.38	12.20	14.21	10.00	19.00	
	Total	89	10.62 ± 3.06	9.97	11.26	5.00	19.00	
Bacterial Count (CFU/10^3^)	Mild	39	16.82 ± 2.87	15.89	17.75	7.00	21.00	0.089
	Moderate	26	15.19 ± 4.33	13.44	16.94	6.00	23.00	
	Severe	24	15.25 ± 2.86	14.04	16.46	11.00	21.00	
	Total	89	15.92 ± 3.42	15.20	16.64	6.00	23.00	
Susceptibility Score	Mild	39	30.26 ± 4.67	28.74	31.77	18.00	39.00	0.211
	Moderate	26	32.08 ± 7.29	29.13	35.02	15.00	42.00	
	Severe	24	33.42 ± 9.46	29.42	37.41	19.00	67.00	
	Total	89	31.64 ± 7.05	30.16	33.13	15.00	67.00	
Circumstance Score	Mild	39	3.59 ± 1.07	3.24	3.94	2.00	7.00	0.001 * Mild < Severe
	Moderate	26	3.27 ± 1.12	2.82	3.72	2.00	6.00	
	Severe	24	5.46 ± 1.91	4.65	6.27	2.00	8.00	
	Total	89	4.00 ± 1.62	3.66	4.34	2.00	8.00	
Chances to Avoid Caries	Mild	39	39.95 ± 6.74	37.76	42.13	27.00	58.00	0.002 * Mild > Severe
	Moderate	26	39.50 ± 9.07	35.84	43.16	22.00	56.00	
	Severe	24	32.71 ± 9.07	28.88	36.54	7.00	53.00	
	Total	89	37.87 ± 8.63	36.05	39.68	7.00	58.00	

One-way ANOVA. Level of significance set at *p* < 0.05, * *p* = statistically significant.

**Table 4:** Distribution of participants according to risk category and severity of intellectual disability.

Risk Category	Severity				*p* Value
	Mild n (%)	Moderate n (%)	Severe n (%)	Total n (%)	
Very low risk	0 (0)	0 (0)	2 (100)	2 (2.3)	0.021 *
Low risk	22 (39.3)	15 (26.8)	19 (33.9)	56 (62.9)	
Moderate risk	17 (54.8)	11 (35.5)	3 (9.7)	31 (34.8)	
Total	39 (43.8)	26 (29.2)	24 (27)	89 (100)	

Chi-square test. Level of significance set at *p* < 0.05, * *p* = statistically significant.

**Table 5:** Comparison of participants’ dMFT and caries experience according to risk category.

Variable		n	Mean ± Std. Deviation (SD)	95% Confidence Interval for Mean		Minimum	Maximum	*p* Value
				Lower Bound	Upper Bound			
Caries Experience	Mild	2	2.18 ± 0.41	1.4363	5.8063	1.90	2.47	0.347
	Moderate	56	1.97 ± 0.33	1.8842	2.0626	1.40	3.15	
	Severe	31	1.89 ± 0.32	1.7801	2.0121	1.35	2.75	
	Total	89	1.95 ± 0.33	1.8820	2.0204	1.35	3.15	
DMFT	Mild	2	10.01 ± 2.83	15.412	35.412	8.0	12.0	0.432
	Moderate	56	11.46 ± 4.51	10.256	12.673	2.0	20.0	
	Severe	31	10.16 ± 4.79	8.402	11.921	1.0	20.0	
	Total	89	10.97 ± 4.59	10.011	11.944	1.0	20.0	

One-way ANOVA. Level of significance set at *p* < 0.05, * *p* = statistically significant.

## Data Availability

Data are available for research purposes upon request from Alzahrani through email: aahalzahrani@bu.edu.sa.
